# Association between Non-Alcoholic Fatty Liver Disease and Dietary Habits, Stress, and Health-Related Quality of Life in Korean Adults

**DOI:** 10.3390/nu12061555

**Published:** 2020-05-27

**Authors:** A Lum Han

**Affiliations:** Department of Family Medicine, Wonkwang University Hospital, Iksan 54538, Korea; qibosarang@naver.com; Tel.: +82-063-859-1301

**Keywords:** non-alcoholic fatty liver disease, health-related quality of life, stress, dietary habits

## Abstract

Considering the increasing prevalence of non-alcoholic fatty liver disease (NAFLD), this study aimed to evaluate the association between NAFLD and dietary habits, stress, and health-related quality of life (HRQoL) in Korean individuals by using data from the Korea National Health and Nutrition Examination Survey (KNHANES) VI 2013–2015. NAFLD was defined in individuals with a hepatic steatosis index (HSI) value ≥36. Eating habits were assessed based on the frequencies of eating and eating out; stress was assessed through the stress perception rate; and the EuroQol-5D (EQ-5D) questionnaire was used to assess the HRQoL. We performed a complex sample logistic regression analysis and estimated the odds ratios by adjusting for significant factors to evaluate associations between NAFLD and dietary habits, stress, and HRQoL. Occurrence of NAFLD was not significantly associated with meal frequencies over one week. With an increase in stress, based on the stress perception rate, the risk of NAFLD increased 1.316-fold (95% confidence interval (CI): 1.175–1.469, *p* < 0.05). Additionally, a decrease in the EQ-5D score by 1 increased the risk of NAFLD 3.38-fold (95% CI: 1.893–4.844, *p* < 0.05). Thus, NAFLD treatment should include stress management, and underlying HRQoL should be considered during treatment.

## 1. Introduction

The incidence of non-alcoholic fatty liver disease (NAFLD) has been increasing globally, including in children and adolescents. NAFLD currently accounts for 75% of the chronic liver disease cases in the United States and its prevalence has doubled in Korea, Japan, and the United Kingdom [1,2,3]. NAFLD has been linked to obesity, various metabolic diseases, and even cardiovascular diseases and mortality. NAFLD occurs independently of alcohol consumption and is associated with an abnormality in insulin sensitivity and fatty acid uptake in the liver, and metabolic alterations mediated by an inflammatory mechanism [2,4]. As with obesity, lifestyle habits, such as lack of exercise and unhealthy diet, have a great impact on the development and exacerbation of NAFLD [1,3,5]. Several studies have shown that consumption of certain nutrients, such as carbohydrates and fructose, affect NAFLD. High intake of carbohydrates, contributing to over 60% of daily energy consumption, or excessive simple sugar intake have been reported to exacerbate NAFLD [3,6,7].

Many studies have also shown that obesity and metabolic disorders can be associated with breakfast habits; particularly when the least of a day’s food is eaten at breakfast, breakfast is skipped or of poor quality, or a nocturnal eating habit affects subsequent breakfast intake [8,9,10,11,12]. Notably, a small breakfast size exacerbated obesity in mouse experiments [8], and nocturnal syndrome was found to be correlated to obesity [9]. Furthermore, consumption of snacks along with skipping breakfast was related to obesity in adolescents [10]. In contrast, a regular diet reduced incidence of obesity and chronic diseases [11] and breakfast has been shown to be important for diet success [12].

Similarly, several studies have investigated the relationship between dietary habits and NAFLD. Poor eating habits, such as high consumption of calories, from carbohydrates and fats, and low intake of vitamins and minerals, are linked to the development of NAFLD [5]. Moreover, use of nutritional supplements can reduce the incidence of NAFLD [13]. In a study that analyzed meal patterns and NAFLD in Korea; traditional, Western and high-carbohydrate, and simple meal patterns were investigated [14]. The traditional eating pattern increased NAFLD, while a simple meal pattern decreased NAFLD [14]. NAFLD patients had a low Korean dietary diversity score, a tool for assessing a balanced diet [15]. However, most existing studies have been performed across different races and countries, and by assessing dietary habits in various ways. As the data on Korean population is limited, there is a need for further studies in Korean patients with NAFLD.

NAFLD is also associated with depression, psychological distress, and psychiatric disease [16,17,18,19,20]. Stress can induce and exacerbate chronic metabolic diseases through excessive release of neurotransmitters such as norepinephrine, epinephrine, hormones such as cortisol, and inflammatory cytokines [18,19,20,21,22,23]. These findings suggest that mental state affects the development of NAFLD through hormones and inflammatory mechanisms. In addition, stress also exacerbates chronic diseases and metabolic diseases through poor lifestyle habits such as overeating, binge eating, excessive alcohol consumption, and smoking [21]. However, studies linking stress with NAFLD are lacking.

The above studies indicate that poor lifestyle, depression, stress, chronic illness, and mortality are associated with NAFLD. Therefore, this provides a good basis for investigating the potential link between NAFLD and health-related quality of life (HRQoL). Several studies report that patients with NAFLD score low on the quality of life assessment tool, in terms of physical, emotional, and economical assessments [24,25,26,27]. The EuroQol- 5D (EQ-5D), developed in Europe, has proven useful as an HRQoL assessment tool in many studies [28,29,30,31], including those involving Koreans [32,33,34].

The hepatic steatosis index (HSI), which is based on levels of alanine aminotransferase (ALT), aspartate aminotransferase (AST), and body-mass index (BMI), is a useful index to diagnose NAFLD that has been validated in various studies [35,36,37,38,39,40]. HSI was reported to have an area under the receiver operating characteristic (AUROC) of 0.81 for the diagnosis of NAFLD [35], which, although a slightly lower AUROC than that of the Framingham Steatosis Index, was of diagnostic value [36]. Another study reported an AUROC of 0.83 for HSI and the tool was deemed to be convenient [37]. Moreover, HSI is cost effective and can be performed before computed tomography (CT) and magnetic resonance imaging (MRI) based diagnoses [38]. In a large-scale cohort study, HSI was found to be an effective indicator for predicting NAFLD in comparison to results using ultrasound [39]. HSI was used in a Korean study that revealed the correlation between NAFLD and asymptomatic hypothyroidism [40].

Based on the results from these studies, we were interested in the correlation between NAFLD, and dietary habits, stress, and HRQoL in a Korean population. We investigated the association between NAFLD, diagnosed using HSI, and these factors using representative data from Koreans.

## 2. Materials and Methods

### 2.1. Study Design

The data are based on a survey by the Korean Centers for Disease Control and Prevention (KCDC) of Korea National Health and Nutrition Examination Survey (KNHANES) VI 2013–2015. Since 1998, KNHANES has conducted a continuous nationwide survey to monitor the health and nutrition of Koreans every year [41]. The survey consists of three components: a health interview, a health checkup, and a nutrition survey. Health interviews and examinations are carried out by trained clinicians. The survey uses a stratified, multi-stage clustered probability sampling. KNHANES has been approved by the KCDC’s Agency Review Board (ARB) [41]. This study followed ethical standards laid out in the declaration of Helsinki. The study was approved by the clinical trial screening committee of Wonkwang University Hospital (ARB approval number 2020-03-012). The KNHANES VI survey was conducted on those aged 19 or older who faithfully completed the survey.

The following persons were excluded: patients with excessive alcohol use (defined as >20 g/day for males and >10 g/day for females); hepatic cellular carcinoma; hepatitis virus B and/or C (HBV, HCV) with systematic history and blood tests; autoimmune hepatitis, drug-induced liver damage, or other liver diseases; liver cirrhosis; or other medical conditions that may affect HSI levels.

**Figure 1:**
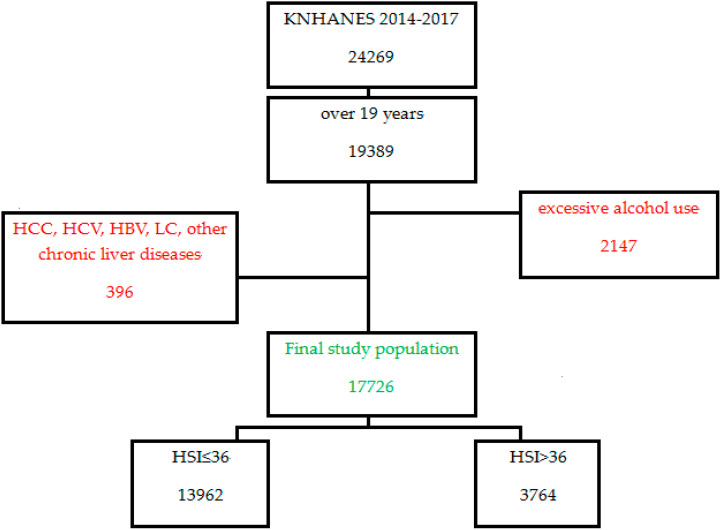
Flow diagram of subjects recruited in this study. HCC, hepatic cellular carcinoma; HCV, hepatitis virus C; HBV, hepatitis virus B; LC, liver cirrhosis; HSI, hepatic steatosis index; KNHNES, Korea National Health and Nutrition Examination Survey.

The subjects were divided into two groups, one group comprising of suspected NAFLD cases and the other a control group (Figure 1). The suspected NAFLD cases were defined as those with an HSI > 36, while the control group had HSI ≤ 36; where HSI was calculated as follows: HSI = 8 × (ALT/AST ratio) + BMI (+2 for women). We used HSI to define NAFLD since the fatty liver index (FLI) cannot be used as gamma glutamyl transferase data are not presented in KNHANES, and ultrasound or CT were not performed.

The usual level of stress perception was investigated with questions that required the individual to respond by selecting one of the following answers: (1) I feel very much, (2) I feel a lot, (3) I feel a little, (4). A person who responded with a (1) or (2) was given a score of 1, and those who marked answers (3) or (4) were scored 0, with 1 indicating high stress and 0 indicating low stress.

HRQoL was evaluated by the widely used EQ-5D [29]. EQ-5D consists of five multiple choice questions and one subjective health level. The health status was determined with regards to five areas: mobility, self-care, usual activities, pain/discomfort, and anxiety/depression. The individuals were asked to choose one of the following three levels: “No problem at all”, “There are some problems”, or “There are many problems.” The responses to the five questions were subjected to the score conversion system, and expressed as a point between 1, which means excellent health, and −1, which indicates health worse than death. The EQ-5D index was then calculated using the following equation:

EQ-5D index = 1 – (0.0081 + (0.1140 × M2) + (0.6274 × M3) + (0.0572 × SC2) + (0.2073 × SC3) + (0.0615 × UA2) + (0.2812 × UA3) + (0.0581 × PD2) + (0.2353 × PD3) + (0.0675 × AD2) + (0.2351 × AD3))

We compared the general factors, stress perception, and EQ-5D values between the normal (HSI ≤ 36) and NAFLD (HSI > 36) groups. We also examined the general factors, cardiovascular-related factors (from blood tests), dietary habits (meal patterns), and whether the individuals had companions at meals in both groups. The relationship between suspected NAFLD and control group and general characteristics, dietary habits, stress, and EQ-5D are presented as an odds ratio (OR). Furthermore, to determine the most important variables in the relationship between the NAFLD and control groups and dietary habits, stress, and EQ-5D, the other variables were corrected, and the correlations are presented as OR.

### 2.2. Anthropometric Measurements

Anthropometric and biochemical measurements were performed by an experienced inspector. The height (cm) and weight (kg) were measured to the nearest 0.1 value, using a Seca 225 instrument (Seca, Hamburg, Germany) and GL-6000-20 scale (G-tech, Seoul, Korea), respectively. BMI was calculated as weight (kg)/square of height (m^2^). Waist circumference (WC) was measured between the margin under the ribs and the iliac crest and recorded to 0.1 cm.

Laboratory tests using blood tests were taken after fasting for 8 h, and samples were immediately transported to a central laboratory (Neodin Medical Institute, Seoul, Korea) for processing. The Hitachi 7600 automatic analyzer (Hitachi, Tokyo, Japan) was used to measure fasting plasma glucose, HbA_1c_ (hemoglobin A_1c_), total cholesterol, triglyceride (TG), high-density lipoprotein (HDL) cholesterol, low-density lipoprotein (LDL) cholesterol, aspartate aminotransferase (AST), and alanine aminotransferase (ALT) levels.

### 2.3. Sociodemographic and Lifestyle Variables

A self-written questionnaire was used to investigate the subject’s sociodemographic and lifestyle variables. Based on the recent smoking status, individuals were divided into “smokers who are currently smoking” (within two years) and “non-smokers.” Alcohol consumption was expressed as grams of alcohol consumed per week. Physical exercise was measured using the International Physical Activity Questionnaire [42]. Regular exercise was defined as three or more times per week and 20 min or more per trial. The education level was divided into four categories: under elementary school graduation, middle school graduation, high school graduation, and above university graduation.

Dietary habits were investigated for the number of breakfasts, lunches, and dinners during a week over the past year and the presence or absence of companions at the time of the meals.

### 2.4. Statistical Analysis

Statistical analyses were performed using PASW (Predictive Analytics SoftWare) statistics software (SPSS version 24.0, IBM SPSS Inc., Chicago, IL, USA). Overall, frequency analysis was performed using the complex sample plan for frequencies analysis. A complex sample Rao-Scott adjusted chi-square test and a complex sample generalized linear model were used to compare general characteristics and dietary habits, stress, HRQoL, and NAFLD. Values are presented as the number (%) or the mean ± standard deviation. A complex sample logistic regression test was used to correlate NAFLD with general characteristics and dietary habits, stress, and HRQoL. We performed a complex sample Rao–Scott adjusted Chi-square test and complex sample generalized linear model t-test (Table 1) to investigate the differences between general characteristics and dietary habits, stress, HRQoL, and NAFLD.

We performed a complex sample logistic regression analysis and estimated the OR and 95% confidence intervals (CI) by adjusting for statistically significant factors (Table 2) to investigate the associations between dietary habits, stress, and HRQoL and potential NAFLD. All data are presented as means ± standard errors (SEs) or percentages (%) for categorical variables. *p* values < 0.05 were considered statistically significant.

## 3. Results

In total, 17,726 subjects (50.6% male; 49.4% female) were recruited in this study, with an average age of 43.93 ± 0.25 years. Table 1 summarizes the differences between the general characteristics, dietary habits, stress perception, and HRQoL among individuals with and without NAFLD. The rate of practice of aerobic exercise was lower among individuals with than in those without NAFLD (41.7% vs. 42.1%; *p* < 0.0001). In a weekly dinner frequency survey over the past year, the rate of almost no daily consumption of dinner was slightly but not significantly lower in the NAFLD group than in the non-NAFLD group. The correlation analysis between EQ-5D and stress perception rate showed a strong correlation with the parameter estimation value of −0.994. In other words, it was found that EQ-5D decreased whenever the stress perception rate increased.

Table 2 delineates the association between NAFLD risk and dietary habits, stress, and HRQoL. The risk of developing NAFLD was higher when skipping breakfast and in the absence of a companion for breakfast (OR: 1.165 and CI: 1.016–1.337, OR: 1.008 and CI: 0.903–1.126, respectively, *p* < 0.05). The NAFLD risk was higher when eating out than when not eating out (OR: 1.250 and CI: 1.006–1.552, *p* < 0.05). Every 1-in increase in WC and an increase in BMI increased the risk of NAFLD (OR: 1.237 and CI: 1.227–1.247, OR: 2.389 and CI: 2.309–2.471, respectively, *p* < 0.05). Every 1-unit increase in FBS (fasting blood sugar), HbA_1c_, total cholesterol, HDL cholesterol, triglycerides, and LDL cholesterol also increased the NAFLD risk (OR: 1.021 and CI: 1.018–1.023, OR: 1.829 and CI: 1.707–1.96, OR: 1.008 and CI: 1.006–1.009, OR: 0.948 and CI: 0.944–0.952, OR: 1.004 and CI: 1.003–1.005, and OR: 1.008 and CI: 1.005–1.011, respectively, *p* < 0.05). Furthermore, the NAFLD risk was higher in the stress-perceiving group (OR: 1.316 and CI: 1.203–1.44, *p* < 0.05). When the EQ-5D score decreased by 1, the risk of developing NAFLD increased (OR: 2.255 and CI: 1.533–3.317, *p* < 0.05).

Table 3 delineates the association between NAFLD and eating habits, HRQoL, and stress after correcting for significant variables. The frequency of breakfast, lunch, and dinner for one week were not significantly associated with NAFLD occurrence. In the stress-perceiving group, the risk of developing NAFLD was higher (OR: 1.314 and CI: 1.175–1.469, *p* < 0.05), and when EQ-5D decreased by 1, the risk of developing NAFLD increased (OR: 3.028 and CI: 1.893–4.844, *p* < 0.05).

## 4. Discussion

This study investigated the association between NAFLD occurrence and dietary habits, stress, and HRQoL among Korean adults. In particular, stress and HRQoL were associated with NAFLD. Herein, the HSI was considered for diagnosing NAFLD, since it is a simple and effective diagnostic index often used among Korean individuals. In a cohort study, a logistic regression model was performed using high serum ALT to serum AST ratio, high BMI, and diabetes mellitus as variables, which are independent risk factors for NAFLD. HSI was shown to have an AUROC of 0.812 [39]. Another study reported that the optimal cut-off value of HSI was 35 (sensitivity, 81%; specificity, 60%) [43]. Several studies have also been conducted considering HSI > 36 to predict NAFLD; we used the same definition in the current study

Numerous studies have investigated the association between NAFLD and nutrient intake, lack of exercise, and carbohydrate intake [44,45,46,47,48,49]; however, few studies have focused on the pattern of dietary habits and NAFLD, or have reported conflicting findings [50,51,52]. To determine whether breakfast consumption influences NAFLD risk, similar to weight loss, it is necessary to assess its association with meal patterns. Herein, the NAFLD risk was lower upon daily breakfast consumption; however, it appeared irrelevant after correcting for the relevant variables, suggesting that total calories, daily carbohydrate intake, and the lack of essential nutrients are better associated with NAFLD than with dietary habits including the frequency of breakfast consumption, dinner consumption, and the frequency of eating out.

The relevance of breakfast in metabolic diseases and obesity has been adequately clarified [10,11,12,53,54]. Furthermore, the frequency of breakfast consumption is low among obese children with a high-energy intake at dinner [55]. The reduction in breakfast consumption in obesity has emerged as a global trend [11]. People do not have breakfast and cannot control their appetite, and so they binge. However, in the current study, the frequency of breakfast consumption and NAFLD risk in Korean subjects were not associated after correcting for related variables. A previous study has examined the composition of meals and NAFLD by investigating the connection between the Mediterranean diet and NAFLD [56]. The study involved 584 subjects with one or more cardiovascular disease risk factors. In the study, NAFLD diagnosis was performed by ultrasound, and adherence to the Mediterranean diet was classified as low, intermediate, or high. The study results indicated that the intermediate and high adherence groups had a lower risk of developing NAFLD than the low adherence group. The study examined the composition of the meal and diagnosed NAFLD using ultrasound, which is different from our study.

Although further studies are required, the following mechanisms could potentially promote the pathogenesis of NAFLD. Consumption of high-energy foods stimulates insulin secretion to regulate blood glucose levels. Insulin resistance results in fat breakdown in peripheral fat tissue to increase the generation of free fatty acids, which eventually increases the intrahepatic influx of free fatty acids. Furthermore, insulin increases fat biosynthesis in the liver, thus influencing the pathogenesis of fatty liver [1,2,3]. There is also a contribution of diet to NAFLD, as opposed to increasing insulin. The results of a mouse study show that intermittent fasting is effective for NAFLD [57]. Intermittent fasting in obese diet mice reduced hepatic lipogenesis and reduced β-oxidation markers to alleviate hepatic steatosis and inflammation.

Herein, the pathophysiological basis is defined as the interaction between psychological status, insulin resistance-related metabolic disorders, and NAFLD [16,17,18,19,20]. Psychological issues are associated with metabolic diseases via the accumulation of visceral fat through hormonal dysregulation, including that of insulin. Psychological factors including stress and depression promote corticotrophin-releasing hormone and cortisol secretion through the hypothalamic-pituitary-adrenal (HPA) axis [16,17,18,19,20]. Dysregulation of the HPA axis leads to the accumulation of visceral fat through hormone secretion and the secretion of inflammatory cytokines like tumor necrosis factor (TNF) [16,17,18,19,20,58]. Inflammatory substances undergo a vicious cycle that causes the relapse of metabolic abnormalities, again through insulin resistance and leptin disruptions [58]. These metabolic abnormalities are a major factor accelerating NAFLD progression [1,2,3]. This hypothesis suggests a direct and indirect link between psychological factors and NAFLD, depending on personal susceptibility.

There have been mouse studies reporting that chronic stress has a negative effect on NAFLD [59]. It was reported that hepatic *TNFα*, *MCP-1*, and *HMOX* mRNA expression levels were high in chronic stress-exposed mice, which was attributed to accelerated liver oxidative stress and inflammatory mechanisms [59]. Studies have revealed that NAFLD is a stress-sensitive disease, as an alteration of the HPA axis exacerbates NAFLD [60,61]. Chronic psychiatric problems are closely related to NAFLD and obesity, and some studies report that psychiatric treatment must be considered when treating NAFLD [61]. This is consistent with the results of the current study, which suggest that stress management is necessary for NAFLD treatment.

NAFLD itself results in other metabolic diseases and is associated with stress and depression. Moreover, its treatment and clinical management are costly [62]. This is speculated to result in the deterioration of the HRQoL in NAFLD, and is supported by our results. Although not presented in a table, additional analysis in the current study showed that EQ-5D had a negative correlation with stress index, which is expected because EQ-5D evaluates various quality of life parameters, including stress.

A previous study has shown that NAFLD leads to economic losses, as shown by health economics, a tool to measure the economic and social consequences of the disease [63]. In addition, various metabolic conditions that can accompany NAFLD can lead to poor HRQoL [64,65,66]. Conversely, people with poor HRQoL are more likely to develop chronic diseases, especially metabolic diseases [64,65,67,68]. These studies, along with our current study, suggest that NAFLD is closely related to HRQoL.

The EQ-5D used in this study as an HRQoL assessment tool has already been demonstrated in Korean studies [69]. EQ-5D was developed by the EuroQoL group for the purpose of clinical and economic evaluation. It is a simple and widely used tool for assessing overall HRQoL. QoL scores have been previously reported in NAFLD including NASH (non-alcoholic steatohepatitis) and cirrhosis in a previous study, which investigated the association between NAFLD and QoL using the Non-alcoholic Steatohepatitis Clinical Research Network and the Short-Form 36 (SF-36) survey [26]. Previous studies investigated the association between NAFLD severity and QoL and reported a poor QoL among individuals with NAFLD [26,62], consistent with the present study. A previous study compared the relative effects of NAFLD on the HRQoL with those of other chronic liver diseases. NAFLD patients had a significantly lower HRQoL than hepatitis B or hepatitis C patients, as revealed through multiple domains of scores of the 29-item Chronic Liver Disease Questionnaire, suggesting that the HRQoL was severely impaired in NAFLD patients [24]. Herein, the EQ-5D indicator was used to evaluate the HRQoL. The closer this score is to 1 point, the higher the QoL [70,71]. Nam et al. (2007) used the EQ-5D to estimate the weightage of the QoL questionnaire, and this indicator was extensively used to assess the QoL among Korean individuals [71]. Furthermore, herein, the probability of NAFLD occurrence was 2.255-fold among the HRQoL indicators after correcting for related variables. Multiple approaches should be considered for the clinical management of NAFLD for improving stress and the HRQoL should be accompanied by classical treatments including pharmacotherapy, lifestyle improvement, and diet.

This study has some limitations. First, since this study included cross-sectional data, it is difficult to explain the causal relationship between stress dietary habits, HRQoL, and NAFLD. Second, the gold-standard diagnosis of NAFLD is liver biopsy and abdominal ultrasound; however, it was difficult to conduct these procedures when surveying the entire population. Furthermore, although the FLI is a commonly used NAFLD indicator, it was difficult to use here because γ-glutamyl transferase levels were not determined. Third, all other variables potentially affecting NAFLD were not considered herein. Despite these limitations, this study has the following strengths. First, this study delineated the importance of psychological aspects by elucidating the association between stress and NAFLD. Second, the number of breakfast, lunch, and dinner meals per week as a dietary habit was not associated with NAFLD. In the future, more specific dietary habits should be investigated to confirm the association. Most importantly, this study shows that the HRQoL is associated with NAFLD among Korean adults. These results may provide essential strategies to facilitate the treatment of NAFLD.

Based on the results of this study, future studies should investigate these associations using more accurate tools for NAFLD diagnosis, while evaluating and comparing HRQoL using various tools. These additional studies may provide evidence for confirming the causal relationship between NAFLD and quality of life.

## Figures and Tables

**Table 1:** Differences between general characteristics and dietary habits, stress, HRQoL, and NAFLD.

		Total (17,726)	HSI ≤ 36	HSI > 36	*p*-Value ^a^
Sex	Male	7986 (50.6)	6004 (47.5)	1982 (61.3)	<0.0001
	Female	9740 (49.4)	7958 (52.5)	1782 (38.7)	
Age	years	43.93 ± 0.25	43.59 ± 0.27	45.1 ± 0.34	<0.0001
Educational level	≤Elementary	4129 (17.3)	3360 (18.1)	769 (14.5)	<0.0001
	Middle	2064 (11.2)	1623 (11.3)	441 (10.8)	
	High	5010 (31)	3868 (30.7)	1142 (32.3)	
	≥College	5753 (35.9)	4515 (35.5)	1238 (37.4)	
Smoking		2905 (19.5)	2110 (18.1)	795 (24.4)	<0.0001
Exercise		6956 (42)	5495 (42.1)	1461 (41.7)	<0.0001
Alcohol		8790 (54.1)	6860 (53.5)	1930 (56)	<0.0001
Stress		4637 (28.2)	3496 (26.9)	1141(32.7)	<0.0001
Breakfast frequency	5–7/week	10,153 (60.2)	8129 (61.1)	2024 (57)	0.002
	3–4/week	1702 (12.3)	1332 (12.2)	370 (12.6)	
	1–2/week	1603 (12.2)	1234 (11.7)	369 (14.2)	
	Rare	1908 (15.2)	1475 (15)	433 (16.2)	
Lunch frequency	5–7/week	13,933 (90.4)	11,074 (90.8)	2859 (89)	0.047
	3–4/week	917 (6.3)	708 (6.2)	209 (7)	
	1–2/week	261 (1.7)	198 (1.6)	63 (2.1)	
	Rare	255 (1.5)	190 (1.4)	65 (2)	
Dinner frequency	5–7/week	13,980 (90.1)	11,079 (90.1)	2901 (90.1)	0.783
	3–4/week	1106 (8.1)	859 (8)	247 (8.3)	
	1–2/week	218 (1.5)	180 (1.6)	38 (1.3)	
	Rare	62 (0.4)	52 (0.4)	10 (0.3)	
Companion for breakfast		7120 (42.3)	5692 (42.8)	1428 (40.4)	0.002
Companion for lunch		10,343 (69.1)	8234 (69.3)	2109 (68)	0.042
Companion for dinner		11,676 (77)	9240 (76.8)	2436 (77.7)	0.485
WC		81.09 ± 0.13	77.84 ± 0.12	92.98 ± 0.17	<0.0001
BMI		23.66 ± 0.04	22.37 ± 0.03	28.3 ± 0.06	<0.0001
Fasting glucose		99.28 ± 0.23	96.71 ± 0.2	108.38 ± 0.6	<0.0001
HbA_1c_		5.61 ± 0.01	5.52 ± 0.01	5.92 ± 0.02	<0.0001
TC		190.3 ± 0.38	187.96 ± 0.38	198.54 ± 0.82	<0.0001
HDL-C		51.11 ± 0.14	52.63 ± 0.15	45.8 ± 0.2	<0.0001
TG		134.18 ± 1.23	120.86 ± 1.32	180.56 ± 2.56	<0.0001
LDL-C		119.07 ± 0.82	114.85 ± 1	124.47 ± 1.27	<0.0001
Stress		0.28 ± 0.004	0.27 ± 0.005	0.33 ± 0.01	<0.0001
EQ-5D		0.95 ± 0.001	0.96 ± 0.001	0.95 ± 0.002	<0.0001

Abbreviations: HRQoL: health-related quality of life; NAFLD: Non-alcoholic fatty liver disease; WC: waist circumference; BMI, body-mass index; HbA_1c_, hemoglobin A_1c_; TC, total cholesterol; HDL-C, high-density lipoprotein cholesterol; TG, triglyceride; LDL-C, low-density lipoprotein cholesterol; Stress, if ‘1’ is recorded for the stress question; EQ-5D, EuroQol- 5 Dimension; HSI, hepatic steatosis index. Definitions: Smoking: the percentage of individuals who are currently smoking (within two years); alcohol, the percentage of alcohol intake more than once a month in the last year; breakfast (lunch and dinner) frequency: breakfast (lunch and dinner) frequency for 1 week in the last year; HSI, hepatic steatosis index = 8 × (alanine aminotransferase / asparate aminotransferase ratio) + BMI (+2 for women); NAFLD, HSI ≥ 36. Values are presented as the number (%) or the mean ± standard deviation. ^a^ The *p*-value was determined through the complex sample Rao–Scott adjusted Chi-square test and complex sample generalized linear model *t*-test.

**Table 2:** General characteristics and the association between NAFLD and dietary habits, stress, and HRQoL.

Characteristics		OR	(95% CI)	*p*-Value ^a^
Sex	Male	1.747	(1.609–1.898)	<0.0001
	Female	1	Reference	
Age		1.047	(1.025–1.069)	<0.0001
Educational level	≤Elementary	0.762	(0.678–0.856)	0.003
	Middle	0.903	(0.788–1.034)	0.190
	High	0.998	(0.897–1.11)	0.660
	≥College	1	Reference	
Smoking		1.349	(1.221–1.49)	<0.0001
Exercise		0.892	(0.821–0.97)	<0.0001
Drinking		1.057	(0.966–1.156)	<0.0001
Breakfast frequency	5–7/week	1	Reference	
	3–4/week	1.104	(0.952–1.28)	0.545
	1–2/week	1.301	(1.123–1.508)	0.245
	Rare	1.165	(1.016–1.337)	0.029
Lunch frequency	5–7/week	1	Reference	
	3–4/week	1.153	(0.946–1.406)	0.312
	1–2/week	1.330	(0.943–1.876)	0.791
	Rare	1.415	(1.005–1.992)	0.046
Dinner frequency	5–7/week	1	Reference	
	3–4/week	1.034	(0.878–1.218)	0.692
	1–2/week	0.833	(0.548–1.267)	0.897
	Rare	0.882	(0.407–1.912)	0.751
Eating out	≥2/day	1.250	(1.006–1.552)	
	1/day	1.103	(0.906–1.343)	
	5–6/week	0.899	(0.739–1.094)	
	3–4/week	0.971	(0.78–1.208)	
	1–2/week	1.008	(0.833–1.22)	
	1–3/month	1.064	(0.868–1.304)	
	Rare	1	Reference	
Companion for breakfast	Yes	1	Reference	
	No	1.008	(0.903–1.126)	0.001
Companion for lunch	Yes	1	Reference	
	No	1.029	(0.932–1.136)	0.018
Companion for dinner	Yes	1	Reference	
	No	0.958	(0.857–1.07)	0.335
WC		1.237	(1.227–1.247)	<0.0001
BMI		2.389	(2.309–2.471)	<0.0001
Fasting glucose		1.021	(1.018–1.023)	<0.0001
HbA_1c_		1.829	(1.707–1.96)	<0.0001
TC		1.008	(1.006–1.009)	<0.0001
HDL-C		0.948	(0.944–0.952)	<0.0001
TG		1.004	(1.003–1.005)	<0.0001
LDL-C		1.008	(1.005–1.011)	<0.0001
Stress recognition		1.316	(1.203–1.44)	<0.0001
EQ-5D		2.255	(1.533–3.317)	<0.0001

Abbreviations: HRQoL, health-related quality of life; NAFLD, Non-alcoholic fatty liver disease; WC, waist circumference; BMI, body-mass index; HbA_1c_, hemoglobin A_1c_; TC, total cholesterol; HDL-C, high-density lipoprotein cholesterol; TG, triglyceride; LDL-C, low-density lipoprotein cholesterol; Stress, if ‘1’ is recorded for the stress question; EQ-5D, EuroQol- 5 Dimension; OR, odds ratio; CI, confidence interval. Definitions: Age, 10-year increase; Smoking, the percentage of individuals who is currently smoking (within two year); alcohol, the percentage of alcohol intake more than once a month in the last year; breakfast (lunch and dinner) frequency, breakfast (lunch and dinner) dinner frequency for 1 week in the last year. ^a^ The *p*-value was determined through the complex sample logistic regression test.

**Table 3:** Association between NAFLD and dietary habits, stress, and HRQoL.

		OR	(95% CI)	*p*-Value ^a^
Breakfast frequency	5–7/week	1	Reference	
	3–4/week	1.052	0.890–1.243	0.925
	1–2/week	1.246	1.053–1.474	0.076
	Rare	1.042	0.890–1.221	0.609
Lunch frequency	5–7/week	1	Reference	
	3–4/week	1.116	0.903–1.378	0.399
	1–2/week	1.291	0.907–1.836	0.891
	Rare	1.333	0.940–1.890	0.107
Dinner frequency	5–7/week	1	Reference	
	3–4/week	1.025	0.852–1.233	0.313
	1–2/week	0.861	0.563–1.317	0.568
	Rare	0.653	0.278–1.533	0.327
Stress		1.314	1.175–1.469	<0.0001
EQ-5D		3.028	1.893–4.844	<0.0001

Abbreviations: HRQoL, health-related quality of life; NAFLD, Non-alcoholic fatty liver disease; EQ-5D, EuroQoL- 5 Dimension; OR, odds ratio; CI, confidence interval. Definitions: Age, 10-year increase; breakfast (lunch and dinner) frequency, breakfast (lunch and dinner) dinner frequency for 1 week in the last year; Stress, if ‘1’ is recorded for the stress question. Adjusted for sex, age, education level, smoking, and exercise. ^a^ The ORs and 95% CI are determined through the complex sample logistic regression test.
